# College of Dentists in England

**Published:** 1857-07

**Authors:** 


### ARTICLE X.

### *College of Dentists in England.*

*Inaugural Meeting.*—This highly important event—the inaugural meeting of the members of the college—took place on the 14th of February; and we do not exaggerate when we say that the proceedings passed off with an *eclát* that even those who best knew the great interest taken in the opening of the college by the great body of the profession, could not have anticipated. The invitations were by cards, sent out in the name of the president, vice-president, and counsel, and signed by the three secretaries. The heads of the college were, very properly, liberal in the distribution of their invitations. They did not confine them to the supporters of the institution, but sent them out widely amongst the profession, not forgetting the members of the Odontological Society. In its desire to have all its proceedings open and above board, it was thought advisable by the council to invite the members of the Odontological Society, through the president and committee of that society; and, accordingly, a sufficient number of cards were forwarded to the president by the council of the college. It, however, appears from a correspondence, which will be found on next page, that Mr. Cartwright returned the invitations; and we have reason to believe that he did so without consulting his council on the subject. We shall not here enter into that matter, which is more properly one for the Odontological Society itself; but we were happy to observe, from the number of members of that society present at this meeting, that if it was intended, by the return of the cards, to keep them away from the inauguration, that object was not achieved.

The meeting took place in the great apartment of the Hanover Square Rooms; all the available space in which was filled by members of the college and visitors long before 8 o'clock, the hour at which the president took the chair. Round the splendid room, which was brilliantly lighted for the occasion, were tables on which were ranged glass-cases, containing interesting specimens of dental phenomena, artificial teeth, operating instruments, &c. At the close of our report, we give a list of the principal matters of exhibition. They were viewed with great interest by the profession; and we are happy to add, that several of them have been presented to the museum of the college.

At a few minutes after 8 o'clock, the president, Mr. J. Robinson, entered the apartment, accompanied by the vice-president, council and secretaries; and was very warmly received by the company.

Having taken the chair, the president called on Mr. Rymer, one of the secretaries, to read the correspondence.

Mr. Rymer then read letters of apology from Lord Stanley, (who expressed his wish for the success of the undertaking,) Mr. W. Coulson, F. R. C. S., Mr. Baron Bramwell, Sir Gardine Wilkinson, Sir W. Ousley, Professor Owen, &c.

The following correspondence was also read:

"*College of Dentists of England,*
"5 *Cavendish Square, London, Feb.* 2, 1857.

Sir—I am requested to inform you, that, by the unanimous voice of the council, cards of invitation to the inaugural meeting of the college (to be held at the Hanover Square Rooms, on Saturday, the 14th Feb. next) will be placed at the disposal of the council of the Odontological Society, for the use of its members. I regret that they will not be ready in time for distribution on the occasion of the society's meeting this evening, but it will afford me much pleasure to forward them on Thursday next, or earlier if possible.

"I remain, sir, yours most respectfully,
"Alfred Hill, *Hon. Corresponding Sec.*

"To *S. Cartwright, Esq.*"

"*Odontological Society, Feb.* 9, 1857.

"Sir—I much regret that sudden and severe indisposition has prevented the president personally replying to your courteous note of the 2nd inst.

"I beg to state, that the secretaries have not been authorised to issue the cards of invitation to the members of the Odontological Society, which you have politely forwarded. I, therefore, return them to you.

"I have the honor to be sir, your obedient servant,

"SAMUEL CARTWRIGHT, *Hon. Sec.*

"To *A. Hill, Esq.*"

Mr. Cartwright's letter elicited marked indications of disapprobation.

The president then delivered the following address; and while doing so was several times interrupted by loud bursts of cheering:

GENTLEMEN: In electing me the first President of the College of Dentists of England, you have conferred upon me the highest and most distinguished honor it was in your power to bestow. The responsibility attached to the office is indeed great; but I cheerfully accept it, because, limited though my individual abilities are, I have the fullest confidence that, acting with your talented vice-presidents, council, and secretaries, I shall be able to do good services in the promotion of the all-important object which our profession has determined to achieve. Gentlemen, at this, the inaugural meeting of the members of the College of Dentists, permit me to congratulate you on the fact that all our proceedings have been public; that the movement in which we are engaged has at no period been sectional; that the entire profession was invited to join in it before your council was even formed, much less invested with power; and that, therefore, the gentlemen whom you have chosen to conduct the affairs of your college during the first year of its existence are, in their official capacity, the elected representatives of the dentists of England. From the moment of our starting up to the present we have had the advantage of free and open discussion. In open assembly, the general laws for the regulation of your body were passed, and your council, secretaries, and other officers elected. Thus have you disarmed suspicion, and day by day gained fresh adherents to your intelligible, and let me add, incontrovertible principles. Some few of our brother practitioners may differ in opinion with the great body of the profession, but I have reason to believe that we have the good will even of those dissentients. At all events, we shall endeavor to deserve it.

No one has attempted to deny that the position of our profession, whether in its relation to the other branches of the healing art, or the public generally, or as affecting our own *status*, was anything but satisfactory. Were it necessary to adduce proof of this, we have it in the large number of practitioners who have sent in their adhesion to the laws which are to regulate our practice, and to bind together the members of our college. Those adhesions show the anxiety of the dentists of this country to attach themselves to an institution based, as this is, upon sound and liberal principles, namely, "self-government and the education of our own members." We have the highest respect for the College of Physicians and an equal respect for the College of Surgeons, but we think that dentists can best be educated by a College of Dentists; at the same time that a prescribed course of independent instruction will be sure to elevate our profession intellectually and socially. A few months since, and we were not a recognised professional community. We cannot much wonder at this when we consider that we were without a head, without a governing body, without laws to guide our practice; but already things have so changed, that our claims to rank with the other branches of the medical profession are universally acknowleged. This, gentlemen, is to be ascribed to the movement on which we have entered, and to the publicity of all our preparatory proceedings. The press has put forward our claims to be recognised as zealous cultivators of our art, and, therefore, entitled to that rank which public opinion has conceded to us. Gentlemen, although requested by the council to prepare an address to the members of the college, it is not my intention to trespass on your patience by giving you an elaborate history of dental science; but I shall, with your kind permission, concisely sketch the progress of the dental art, with a view of placing before you our position as a co-ordinate branch of the healing craft. To do this, it is not necessary to determine the exact date in which the practice of medicine was first known. Like many other arts, its origin is involved in considerable obscurity; but this we know, that, at a very early age indeed, the power to heal the sick, mitigate the pangs of suffering humanity, and stand between disease and death, was esteemed to be a god-like attribute. Much has been said, and probably more written, respecting the antiquity of medicine. Now, gentlemen, I claim precisely the same antiquity for dental art. The ancients, who inclined to mythological rather than to natural causes, affirmed the science of medicine to be a divine emanation, and impersonated it, first in Apollo, and next to Æsculapius. Thus its early history is mixed up with mythology and poetry. Although it is impossible to imagine a state of primitive society so happy as to be free from pain and disease, yet we find that in proportion as people advanced towards civilization, and abandoned their more simple habits of living for idleness and luxury, so did disease in its various forms call into requisition the skill of the physician. Those same habits of refinement and luxury, and the consequent attention to personal appearance, must have rendered the practice of the dental art one of considerable importance even at a very early period of the world's history. According to Herodotus, there was a subdivision of medical science, and no practitioner was allowed to practice any but his own peculiar branch. Thus, some were occulists; others attended solely to diseases of the head; and, others again, to those of the teeth. It is not, however, till the time of Hippocrates that we meet with any distinct notice of diseases of the teeth. This appears the more extraordinary, as the significance of these organs—to say nothing of their useful or ornamental functions—was regarded by the ancient Egyptians in a very remarkable manner. One of their most severe and degrading punishments consisted in the abstraction of a front tooth. There can, therefore, be, I think, little doubt that the manufacture of artificial teeth and other branches of the dental art were practiced at a much earlier date than that of which we have the first mention in history. The loss of a front tooth, whether by disease or otherwise, would, during the existence of that Egyptian punishment, have given rise to unpleasant suspicions; and it may be presumed that every exertion would have been made to supply the deficiency. Belzoni and others discovered rudely manufactured teeth in the sarcophagi of the Egyptians. Again, as regards the use of gold leaf, Sir Gardiner Wilkinson observes as a singular fact, that the Egyptians stopped teeth with gold—a method of stopping not very long practiced in Europe. Proof of this has been obtained by the examination of some mummies from Thebes. We have historical evidence that the general appearance of the teeth, and their diseases, attracted considerable attention amongst the Greeks and the Romans. The wearing of artificial teeth formed the subject of satire for some of the poets. I could even, if necessary, enumerate many other circumstances to show that dentistry engaged as large a share of the attention of the ancients as did any other branch of the healing art—a circumstance to be attributed to that subdivision of medical practice to which I have before referred. Hippocrates and Galen mention sundry electuaries for beautifying the teeth, but describe nothing that may be called the proper art and science of dentistry. Albucases, an Arabian physician, who lived in the early part of the eleventh century, wrote on diseases of the teeth, and gave drawings of a number of instruments then in use for extracting, scraping, and other dental operations. He, moreover, gives instructions as to how teeth should be extracted, and directs that, if hollow, they be stopped with cotton—refers to filing teeth, and to fastening loose ones with *gold thread*. Ætius details a variety of applications for removing teeth without an operation; and it is worthy of observation that one of these applications actually contains red arsenic. This substance, as you are all aware, was introduced into our practice only a few years ago as an escharotic for destroying the dental pulp previously to stopping!

"We think our fathers fools, so wise we grow,
Our wiser sons, no doubt, will think us so."

The red arsenic was supposed, gentlemen, to be a modern discovery; but it appears to have been very well known to some of those old gentlemen whom we, in our wisdom, are sometimes disposed to think very lightly of. However, giving the ancients all the credit to which they are entitled, it is not at all extraordinary that some of their opinions should be useless for our purposes. They had not the advantage of that mighty power, the microscope. At least no mention is made of microscopic investigations in those times to which I have been alluding. It is somewhat difficult to appreciate the observations of Hippocrates, who describes the teeth as glutinous extracts, from which the fatty matter has been burnt up by heat, and affirms that they are harder than the other bones, because they have no heat in them. Aristotle, who has some excellent observations respecting the teeth of men and animals, declares them to be the only bones which grow through the whole of life, observing that if they did not, that they would soon be worn away by attrition. He adds, that the growth is manifest in those teeth that have lost their corresponding opposites in the other jaw—referring, of course, to the elongation of teeth arising from a want of an opposing force. By these few observations from ancient authors, I think I have clearly shown the antiquity of our calling; the value and importance of the teeth, as adjuncts to health and appearance, and the attention bestowed upon them at the earliest periods of medical history. I shall, however, proceed, and bring my observations somewhat nearer to our own time.

At the end of the 16th century, the dental art began to receive that peculiar attention to which, from its importance, difficulty, and general usefulness, it is eminently entitled. About that time there were no less than thirty-eight treatises published on the subject; and although their usefulness has been greatly diminished by discoveries since made, still they are highly interesting as evidence that dental surgery was in the 16th century considered of very great importance, and that time and experience only were required to raise it to its proper station in medical art. The first attempt to classify diseases of the teeth was, in the 18th century, made by M. Fouchard, who has justly been denominated the father of dental science. Previously to his time, the practitioners appear to have merely considered the teeth in their mechanical arrangement, taking little or no account of them as complex organic structures, entering by their own vitality into the formation of the living body. M. Fouchard had not only the merit of directing the attention to the construction and separate treatment of the teeth, but he also pointed out the indications which, in common with the adjacent parts, they furnish of the general state of health. It was, unquestionably, a most important advancement in dental science to demonstrate that the teeth afford an indication, not merely of the apparent, but also of the innate and fundamental constitution of individuals. It was, however, only after 36 years, of close study that Haller published, in 1747, his remarkable work on physiological and pathological science, the results of actual observation of the laws that govern the growth and decay of living bodies. In the mean time, rapid and valuable improvements were being made in the mechanical department of our profession. So much had the subject grown into consideration, that by the end of the 18th century, no less than 158 works had been published on the subject, including those of Malpighi, Purkenji, Retyius, Muller, &c. The first work that appeared in England, in a popular form, was by Berdmore, and was published in 1770. In 1772, the great John Hunter did not consider it *derogatory* to his position and reputation to give the profession the result of his dental investigations, in his celebrated work on the *Natural History of the Teeth.* This was followed by the *Inaugural Disssertation on the Structure of the Teeth of Men and Animals*, by Robert Blake, in 1798. Time will not permit me, on this occasion, to enumerate the various valuable contributions to dental science that have since been published; but I may, without wishing to lessen the merits of other gentlemen, mention the names of Fox, Bell, Goodsir, Nasmyth, Tomes, and Professor Owen, as those of men who have become prominently identified with our profession, and who stand pre-eminent as physiologists and pathologists. I must also direct your attention to some of our transatlantic brethren, who have likewise done much in the walks of dental physiology and pathology, and dental mechanism.

And here I would ask you to bear in mind that the dentists of the United States have, for some years, held, as a body, a very different *status* to that occupied by the dentists of this country; but what is still more worthy of your remembrance of this—for us, hopeful fact—that those who first attempted to found a college of dentists in America, had to encounter precisely the same amount of difficulty as that which presented itself to the gentlemen who commenced our present undertaking; yet, in spite of all obstacles, educational colleges for teaching the dental science, both surgical and mechanical, have been firmly established in America—established, be it understood, in entire independence of any college of physicians or surgeons. These dental colleges not only have an independent curriculum of special study, but, connected with them, are dental hospitals and dispensaries, which are generally attached to the colleges. The pupils are systematically taught every branch of the dental art, by a course of study that embraces dental anatomy, physiology, special pathology and therapeutics; chemistry and metallurgy, the principles and practice of dental surgery, operative dentistry, and dental mechanism. Having gone through the prescribed course of study, the pupils are examined by the professors in each department, and if the examinations be passed in a satisfactory manner, a college diploma is granted, which authorises the holder to assume the title of "Doctor of Dental Surgery." A vast deal has been said about our wishing to adopt the *modus operandi* of the dentists of the United States—that which I have just briefly described. Now, gentlemen, I confess my inability to perceive how we lower our dignity, either individually or collectively, by endeavoring to form an institution similar to those which have not only fulfilled the object for which they were more immediately designed, but have raised the position of the dental profession generally. It appears to me that ours is not so different from every other profession, as that we do not possess within our own body sufficient knowledge of the theory and practice of our art, to enable us to educate and examine our own pupils, as dentists, without having a recourse to a college whose province it is to grant diplomas to

*pure* surgeons only. In my opinion, *we, the dentists of England*, can ourselves best carry out the great objects which all legitimate members of the profession have in view; and these I may in a very few words sum up. They are, to raise our *status*, to give professional skill its rightful pre-eminence, and to crush that *charlatanism* which has, in too many instances, succeeded in preying upon the public health and purse, under cover of a pretended knowledge of the theory and practice of dentistry. Unquestionably, the dental art has progressed in the United States. This may, in no small degree, be attributed to the energy of character for which Brother Jonathan is so renowned; but it must principally be attributed to that absence of small jealousies and invidious distinctions in reference to less fortunate brother practitioners, which allows of a free communication of ideas amongst all members of the profession, and which has brought to a successful accomplishment, the work of establishing associtions for the protection and furtherance of the common interests.

The present movement arose from a conviction on the minds of many dental practitioners, that the time had arrived when the study of our science should be put upon the footing which ought to be occupied by a study which had engaged the attention of Hippocrates, five hundred years before the Christian era, and has ever since found numerous advocates, including the modern Hippocrates, the renowned John Hunter. When the movement was originated, we really had not the *locus standi* of a recognized profession. Neither had we any common bond to link us together for the advancement of the dental art. Each member did the best he could to further his own ends, and viewed the exertions of his brotherhood through a contracted medium, if not with the jealous feelings of envious rivalry. But what a change has already been effected! What a noble spirit of liberality towards each other now characterizes our meetings! We no longer fear the wholesome competition that is sure to arise from the elevation of our profession. Liberal fundamental laws have been laid down, and a liberal interpretation has been given to these laws, in order that no dentist really qualified and willing to uphold the respectability and dignity of his profession may be excluded. There have been no nice lines of demarcation—no paltry distinction; and hence it is that our advance has been rapid, and that we have obtained the support of the great body of the profession.

I feel that it would be unseemly in me to conclude my inaugural address without, in your name and my own, paying the slight tribute of acknowledgment to the gentleman who were the promoters of this great undertaking, Messrs. Rymer, Hill, Mackenzie, Perkins, and the provisional committee generally. I offer them my own, and I think I may add your sincere thanks, for their indefatigable exertions, and for the zealous and judicious manner in which they brought together so large a number of our hitherto divided profession, and thus form the nucleus of the present society. To members and associates, I would express a hope that they will, one and all, prepare interesting papers on professional matters, so that we may all be benefitted, enlightened, and improved by associating together. The gentlemen who have so kindly consented to deliver before us the preliminary course of lectures for the present session, I thank most sincerely; and I earnestly commend those lectures to the members, associates and pupils, as being in every way worthy of their attentive study. If diligently attended to from the commencement, they will be found to form a very solid foundation for those studies which are to follow, and which are intended to give such an elevation to our professional character as shall command, for the diploma of the College of the Dentists of England, the respect of this, and every other country wherein dental science is practiced.

Dr. Roberts, of Edinburgh, then rose and said: Gentlemen, allow me to propose, which I do with very great pleasure, a cordial vote of thanks to our President, for the valuable, the interesting and the eloquent address which he has just delivered [hear, hear, and loud cheers.] To that able address we have all listened with the greatest pleasure; and I am sure you will agree with me, that it deserves our warm acknowledgments [renewed cheers.] You will also feel that Mr. Robinson merits our best thanks for the great energy he has displayed, and the influence he has brought to bear on the establishment of the College of Dentists of England [hear, hear, and applause.]

Mr. Bate, of Brighton, seconded the motion, which passed with acclamation.

The President returned thanks; and read a list of the different introductory lectures to be delivered in the ensuing session. The announcement of these lectures was received with loud cheers.

The company then partook of refreshments, and afterwards spent a considerable time in examining the various objects of interest with which the walls were covered, and the glass cases along the tables stocked.

Amongst those who exhibited on the occasion, were:

Mr. Weedon.—A collection of extracting forceps, scaling and stopping instruments; instruments for microscopical preparations.

Mr. Everard.—Two cases of adjusted forceps, eighteen in each case.

Mr. Dixon.—A new and very ingenious drill, working at right angles, for the molar teeth; a new file carrier forceps for the posterior teeth; condensing forceps with shifting bits, employed in consolidating gold fittings; a large variety of stopping instruments; a collection of extracting forceps; a very ingeniously contrived instrument case for traveling, fitted with drawers, containing forceps, stopping instruments, scalers, &c.

Messrs. Ash & Sons.—A fine collection of the various mineral teeth, a new portable lathe, various stopping instruments, extracting forceps, double pin nippers, corundum files, new copper frames for soldering flat teeth, specimens of gum teeth, mounted, &c.

Mr. W. Harnett.—Specimens of prepared gutta percha, colored and white; gold foil corundum, in its native state and in wheels; sponge gold pluggers; flat teeth with platina backs; tube molar teeth pierced for swivels; an ingenious machine for cutting mineral teeth after manufacture.

Mr. Pratt.—Three cases containing corundum wheels and files; samples of gold leaf; gutta percha rubbers; gutta percha, pure and tinted; metallic filings, and two teeth stopped with *his* gold leaf.

Mr. Taylor.—A case containing a very superior collection of his mineral teeth.

Messrs. Smale Brothers. An interesting collection of preparations, showing the facial nerves and arteries in wax; various skulls exemplifying the stages of dentition; beautiful and rare specimens of the hippopotamus tusk; one of them nearly annular; a bar of aluminium; an articulated skull, one side exhibiting the arteries and veins colored, the other the nerves. This skull was beautifully prepared, and showed the interior of the petrous portion of the temporal bone; the ethmoid and sphenoid also appeared perfect.

Mr. Mackenzie.—A fine collection of minerals, used in the manufacture of artificial teeth.

Mr. Brown.—The skull of the champanzee; also a cast of the mouth.

Mr. Rymer.—A lower molar with four fangs.

Mr. Mouseley. (Hull.)—Osseous union of the temporary lateral and canine (presented to the College.)

Mr. Adam Thompson.—Three beautiful preparations exhibiting the teeth in infancy, youth, and at full age, which he has generously presented to the museum of the College.

Mr. Jacob.—A newly invented hand drill stock for excavating caries.

Mr. Harley.—A rare specimen of salivary calculus upon a piece of artificial bone work in the anterior part of the lower jaw (presented to the College.)

Mr. Parker, (Birmingham.)—A capital collection of models of irregularities of the teeth; others, showing the various stages of the development of the dentes sapientiæ.

Mr. Williamson, (Leicester.)—Pathological specimens; one of an upper molar tooth, in which exostosis is clearly defined on the three fangs; another, a portion of the upper jaw of a child, exfoliated from the injudicious use of mercury; two rare specimens of exostosis and absorption combined in the inferior molars, both extracted from the same jaw.

Mr. Hepburn.—Several extraordinary models of congenital cleft palate.

Drs. Fowler and Preterre, (Paris.)—A highly ingenious mechanical substitute for the inferior jaw, (which had been entirely removed,) mounted with Allen's process; the lower substitute was attached to an upper palate plate by movable hinge joints as well as with spiral springs. This is one of the most interesting and novel pieces of mechanical work we have ever seen, and has been of incalculable benefit to the patient. It excited much interest at the meeting. These gentlemen also exhibited an enormous cyst at the symphysis of the inferior maxilla which had been removed, and in the interior could be discovered the exciting cause, the eruption of the left lower canine lying horizontally in the cavity of the cyst.

Mr. Underwood.—A fine specimen of salivary calculus upon the labial surface of a second molar in the upper jaw; several very interesting models of irregularities; osseous union of deciduous teeth; cases of necrosed teeth, arising from mechanical injury and disease; some teeth found at Pompeii; exfoliation of a large portion of the alveolus of a child containing the embryo of several permanent teeth.

Mr. W. Bennett, (Associate.)—Very choice specimens of plaster of paris (gypsum) showing the different stages of calcination.

The President.—The metals used in dentistry, from their pure to the manufactured state; specimens of all the minerals used in the manufacture of artificial teeth; also a great variety of metallic oxyds used in the enamels; a model of an American furnace for baking mineral block teeth; the *Materia Medica* of dentistry, specially prepared by Messrs. Morson & Son; a large number of casts, showing the different irregularities of the teeth, one in particular exhibited two supernumerary teeth resembling bicuspids, developed between the central and lateral in the superior jaw; a fine specimen of the osseous union of the second and third molar in the superior jaw; several osseous unions in the temporary teeth; a fine preparation of exostosis in the inferior dens sapientiæ; exfoliation of the alveolus of the lower jaw of a child, containing five of the permanent teeth; a curious development of an inferior dens sapientiæ in the coronoid process, and a large variety of other preparations illustrating dental pathology, &c.

Mr. Hutchins.—Specimen of osseous union of the permanent central and lateral in the upper jaw; exostosis at the fang of lower bicuspid; exostosis at the neck of an upper canine, the deposit having partiallycovered the anterior surface of the enamel.

Mr. Jabez Hogg.—A large number of beautifully executed diagrams, illustrating the development of the teeth and their structure, as seen under the microscope. Mr. Hogg's valuable microscopes (manufactured by Baker) were exhibited upon a platform; and this gentleman kindly lent his large collection of sections of the teeth, and personally explained their structure.

Mr. Baker also exhibited several of his microscopes.

Mr. Purland exhibited two cases; the first contained fine specimens of the canine tooth of Bengal tiger, in section, showing dental pulp. Lower incisor of African lion. Molar of rhinoceros, in section. Upper canine tooth of alligator, in section, from India. Incisors of heifer, in section. Incisors and molar of bull, whole and in section. Incisors, canine, bicuspids, and molar of lamb. Incisors, canine, bicuspids, and molars of sheep, whole and in section. Teeth of pig, whole and in section. Horse, crown of molar, and molar in section, from Waterloo. Upper and lower jaw of a monkey. Lower jaw, and upper set of teeth of a fox. Section of jaw of sperm whale, and tooth of sperm whale, in section. Sections of elephant's tusk. Section of hippopotamus tooth. Section of walrus tooth. Teeth of shark—antediluvian—very fine.

The second case contained a fine collection of human teeth. Sections of central, lateral, cuspid, bicuspid, molar, and sapientiæ molars, ordinary and extraordinary size, upper and lower. Very extraordinary lower incisors, lateral, and canine teeth. Canines with two and three fangs. Bicuspids with three fangs. Lower molars with three fangs. Upper set of infant's teeth. Staminæ of permanent set. Eleven varieties of dens sapientiæ, from small to very large. Sapientiæ with one, two, three and four fangs. Sapientiæ of very remarkable formation, fangs at right angles, &c. Supernumerary teeth taken from the palate. Nerves of centre, lateral and canine, displayed. Eight specimens of the formation of salivary calculus deposited upon the teeth. Eight specimens of salivary calculus, removed from the teeth. Dens antiqua—Egyptian, British, Roman, Anglo-Saxon, Danish. Eleven teeth and stumps extracted from one patient, in May, 1845, whilst in the "mesmeric trance."

There were many other contributions, which our space will not allow of our noticing.

*Monthly Meeting.*—The first ordinary meeting of the members of the college was held on Tuesday evening, March 3d, for the reading of papers on professional subjects. The chair was taken by the president, who called on Mr. Rymer to read a list of twenty candidates for admission at the next ballot for members. Mr. Fox then announced the receipt of fifty-three contributions to the museum, including specimens of the human teeth, skulls of fish, early specimens of mineral teeth, anatomical preparations relating to the teeth, ancient tooth forceps, &c. The names of the donors are Messrs. Harding, Hockley, Mackenzie, Hill, Fox, A. Thompson, Dubois, Rymer, Austin, (Liverpool,) Brindley, (Sheffield,) Mosely, (Hull,) West, and Cocking. Mr. Rymer also read a long list of valuable contributions to the library, from Messrs. Ash, Dubois, Purland, and Fox. At the conclusion of the report, the president expressed his thanks to the donors for their liberal contributions, and announced that Mr. Mackenzie would now read a paper.

Mr. Donaldson Mackenzie then read a paper upon that portion of mineralogy which bears directly upon the production of mineral teeth.

He introduced the subject by remarking, that the college movement being essentially an educational one, he had prepared a paper on the properties of minerals, with a view of inducing the attendance of the junior members of the profession, and advocating the necessity of every dentist acquiring a knowledge of the subject. Half a century since, the study of the science of mineralogy would have been looked upon as superfluous, or perhaps useless; but now, judging from the specimens of mineral work exhibited in the lecture room, such vast strides had been made towards perfection, that it was not now safe or right for any dentist to be ignorant of the science; and he had no doubt but as our knowledge of mineralogical chemistry advanced, perfection in mineral works would follow in a proportionate degree. Indeed, he could not see how excellence could be attained in any undertaking without an acquaintance with the nature of the materials employed. After a few remarks upon the life of the late Mr. Wedgewood, and his inventions applicable to the profession, as introductory to this subject, Mr. Mackenzie gave a slight geological sketch of the crust of the earth and the formation of igneous and stratified rocks, showing that almost all the materals necessary to compose artificial mineral teeth, were to be procured from the granite, either as a constituent of that rock, or as accidentally incorporated into its substance; that although the process of induration of rocks never took place upon the surface of the earth, namely, on to that portion subject to the influence of the atmosphere, which is not covered with water—yet that the preparatory process of sedementary rock-making did; and it was to this disintegrating influence, that we were mostly indebted for the means of discovering those minerals which were most useful in the teeth-making. After a few words relative to the mode pursued by the Chinese in their china manufacture, he described the mineral kavlin, its mode of occurrence, locality, and use to the profession, also its base, aluminium; explaining how much the dentists had been disappointed in their hopes of finding this metal an auxiliary, which is now produced in large quantities from the Greenland mineral cryolite, showing it to partake of the nature of metals perfectly unfit for any purpose the dentist could adapt it to. The next mineral described was vitrious and calcedonic quartz, quoting the disquisitions of the learned upon the igneous origin of the former, and the aqueous origin of the latter; Mr. Mackenzie entering rather warmly into the curious speculations which exist, regarding the origin of flint, which he said was not yet satisfactorily defined, and was still open to investigation—

one replete with interest to the inquiring mind. Mr. Mackenzie next drew attention to the mineral felspar, pointing out its varieties, their local appearance, chemical equivalents, also their individual and combined uses to the dentist.

This was followed by a discourse upon the minerals talc, mica, and asbestos; describing their peculiarities, localities, appearance, and general uses to the dentist, as improving the tone and quality of mineral teeth; he likewise alluded to their domestic appliances. With these, he concluded the category of minerals made use of in the substance or body of the teeth; made some remarks on the native oxyds of titanium, uranium, crome, gold, platina, iron, copper, cobalt, &c. Next came under review, and were examined *seriatim*, their individual action upon mineral substâances, the modes of obtaining the various shades of color, and also the preparation of the artificial oxyds themselves.

When Mr. Mackenzie concluded, the president thanked him for the very interesting scientific paper he had read to the meeting, and on which he (the president) invited discussion.

Mr. P. Matthews requested to know whether in the manufacture of his teeth, Mr. Mackenzie had ever mixed his oxyd of gold in conjunction with felspar?

Mr. Mackenzie replied in the affirmative; and observed, that when mixed with felspar, the oxyd was rendered more fusible.

Mr. Perkins wished to be informed what proportion of the oxyd of iron to the oxyd of gold would Mr. Mackenzie recommend.

Mr. Mackenzie regretted that he could not, at the present moment, remember the proportions.

Mr. Purland repudiated the idea of the Chinese having been the first to manufacture teeth; and referred to numerous specimens in the British museum, manufactured between 4000 and 5000 years ago, by the Egyptians, who he considered were the original makers.

On the subject of flint, Mr. Purland said, he had discovered pieces of wood in the centre; and remarked upon the artificial teeth he had found in mummies.

Mr. Weiss inquired whether arsenic had ever been employed in the manufacture of porcelain teeth?

Mr. Mackenzie observed, that this substance would not stand the great heat.

Mr. Matthews remarked upon the large quantities of silica found in hay, straw, and glass. In the former it was found absolutely in a pure state.

Mr. Hill wished to know the probable cost of the various materials employed in the manufacture of teeth?

Mr. Mackenzie would, on a future occasion, give some explanations on that subject.

Mr. Rymer wished to know which were the best works for obtaining the requisite knowledge of these various substances?

Mr. Mackenzie stated he had found a small work by Varley, published by Weale, to be as good as any upon the subject.

Mr. Matthews stated that he had had considerable experience in the manufacture of teeth, and he was desirous of knowing how much should be allowed for contraction during the baking process.

Mr. Mackenzie: The contraction was, in the first instance sufficient to crack the material, which was afterwarde filled up, and again submitted to the process.

Mr. Williams remarked that he had found the contraction so great, that when a piece of work had been submitted to the baking process, it did not afterwards fit the model.

Mr. W. Bennets (Associate) remarked that he had found from practical experience, that the best model to stand the heat of the furnace without contraction, and upon which the platina plate was placed during the baking, was composed of asbestos, plaster of paris and sand.

Mr. Bell had had considerable experience in the matter, and he found that the contraction amounted to one-fifth.

After a few observations by Mr. Harley and Mr. Perkins, Mr. Mackenzie described the process of making a piece with mineral gum, observing that on some future occasion he would endeavor to illustrate this part with suitable diagrams of the furnaces, ruffles, &c.

The president observed that as they had not at present any laboritory, furnaces, &c., in which to give practical demonstration, he and his partner would be very happy to afford the use of their own to show the process of enameling to any student so inclined. The meeting, he was quite sure, would cordially agree with him in thanking Mr. Mackenzie for the interesting and instructive paper he had been kind enough to read.

Mr. Matthews then moved, and Mr. Purland seconded, a cordial vote of thanks to Mr. Mackenzie, which was passed with applause. This terminated the business of the meeting. The members and their friends then examined the various collections of minerals and metallic oxyds used in the manufacture of teeth; specimens of sets and partial sets of teeth manufactured from fifty to sixty years back; also the various specimens of single mineral teeth belonging to Mr. Purland and others.

*Dr. Steggall's Lectures.*—On Thursday, Feb. 19, Dr. Steggall delivered the first of his course of four lectures on "Descriptive anatomy," to a large number of the members and associates of the college. The lecturer observed, in commencing, that he was not prepared to see such a large audience; neither must they expect that he could do justice to his subject in four lectures; but as this was considered by the council to be more of an introductory course, he would endeavor to compress as much as possible into each of the four.

In the course of this and his three subsequent lectures, Dr. Steggall treated his subject in detail, giving the student a remarkably clear exposition of the structure of the head and jaws, and the primary and permanent teeth, muscles of the mouth, &c., &c.

In his fourth lecture, the doctor drew attention to the arteries, veins, and nerves, dwelling particularly on the external carotid and its branches, the superior thyroid, lingual and facial; the occipital and auricular; the inferior pharyngeal and the temporal and the internal maxillary. He then described the cerebral nerves, dwelling particularly on the second and third branches of the fifth pair, viz. the superior and inferior maxillary nerves and their connection with the teeth.

On the whole, his course of lectures formed an admirable introduction to the studies which the council has determined to make a *sine qua non* for all who propose to graduate in the College of Dentists.

At the close of his fourth lecture, Dr. Steggall was greeted with loud and long continued applause; and

The president observed that they were much indebted to Dr. Steggall for the kind and liberal spirit he had displayed in delivering these introductory lectures. All present must be aware how difficult it must have been to compress into four lectures what ought to occupy some twenty or thirty. They begged to thank Dr. Steggall, and when they again met next session, they hoped to extend the course to some twenty-five or thirty lectures upon this important branch of study. He must also thank Mr. John Harnet, for his kindness in having lent the beautiful anatomical preparations for illustrating the lecture.

The *Medical Circular* thus alludes to Dr. Steggall's lectures: "On Thursday evening, March 12th, Dr. Steggall concluded his introductory course of lectures on the descriptive anatomy of the head and face, to the students and members, at their lecture rooms, 5 Cavendish square. Dr. Steggall is a careful and lucid demonstrator, and will prove a valuable teacher to the students of the College, should they be fortunate enough to secure his services as anatomical and physiological lecturer for the future. The short course of lectures were well attended, and from the quiet earnest attention displayed by the auditory, it is manifest that the desire to acquire professional knowledge is thoroughly awakened. The lecturer and students have been much assisted by the kindness of Mr. John Harnet, who, in the handsomest manner, has supplied them with many beautiful osteological preparations, which enabled the lecturer clearly to demonstrate his subject, and the students to follow his course with ease."

*Mr. Jabez Hogg's Lectures.*—Mr. Hogg, surgeon to the Royal Westminster Opthalmic Hospital, delivered his first of a course of three lectnres, on Thursday, March 19. The lecture hall was crowded by members and associates. Around the hall was a fine collection of diagrams, &c., &c. The subject of this very interesting lecture, which was listened to with the most marked attention was, "histological anatomy," its value to the student; the microscope, historical introduction, optical arrangements, appliances, and preparation of objects.

*Mr. G. Simpson's Lectures.*—A course of lectures on "general and special anatomy," will be commenced by Mr. G. Simpson, F.R.C.S., on the 2nd inst.

---

### *The College of Dentists of England: its Origin and Development.*

(Communicated.)

In commencing the record of the reformatory movement in the dental profession, bearing the above title, it is both natural and pleasant to revert to its real origin, and notice the elements from which it sprung—to trace backwards, step by step, the progress of the scheme, which, within a few months, has grown so steadily, and at the same time so firmly into a definite form.

If we look at the College of Dentists of England, primarily, a recognized institution for the advancement of dental science and art, and secondarily, and as a matter of sequence, a guarantee to the too long plundered public, we see a council, with its presidents, vice-presidents, treasurer, and secretaries, the necessary office bearers to provide for its efficient working, a long list of members, a complete set of lectures for the first session, and the nucleus of a museum and library; in all a goodly array with a certain solidity of purpose about it, which bids fair to stand the test of divided opinions as to its capacities. This we are compelled to acknowledge, while knowing the materials that such energetic pioneers will have to clear away.

A short time since, and this substantial fabric was to be comprehended in the persons of some eighteen practitioners, who under the name of provisional committee men, were content to labor at the oar, in order to bring about the best solution of difficulties which had met them, in their endeavors to carry out certain duties entrusted to them.

Another step backwards, and the interest of the undertaking was embodied in about half a dozen gentlemen, who had met to cogitate over the best manner of conducting the first public meeting of the profession, then in view, and to decide upon the method to be employed in bringing about the much desired end.

One more step to the rear, and we find the determination to arouse the apathy of the profession, by convening an open meeting, engendered in the mind of a young practitioner, residing away from the great theatre wherein the piece was to be performed, but engendered nevertheless with a full persuasion that perseverance would prevail.

Thus we prove, that as in many other movements of a similar nature and design, *one* person simply deserves the credit of originating a scheme, the benefit of which will soon be felt in many directions. To Mr. Samuel Lee Rymer, of Croydon, then, is due the commendation of first *practically* introducing the idea of forming a Dental College in London. It is not asserted that Mr. Rymer first thought such a thing possible; as doubtless, many other worthy members of the profession had conceived the same notion, and it is well known, that in some crude form or other, most of those gentleman who desire to adorn, rather than disgrace the profession, had hoped that the future would bring forth some institution or association, which might be made available for the perfecting of their acquirement, both in the surgical and mechanical departments. But "*faetis non verbis*" appears to have been the motto which Mr. Rymer chose to adopt. Whilst other men contented themselves with thinking the matter over, or at most, putting their thoughts to paper, he dared the danger of defeat, and leaving off *thinking* and commencing *acting*, laid the foundation of this edifice at his own risk. Here then lies his praise: to this fact his name will ever be attached; and for this effort, he certainly deserves the thanks of every gentleman in the profession, who desires to see his chosen calling wrested from the gripe of the unprincipled and unqualified practitioner.

That Mr. Rymer had thought and pondered over the possibility of establishing an institution of this kind and calibre, may be gathered from the fact, that in August, 1855, he published his views on the necessity of reform, and laid down lines of conduct which he considered it advisable to pursue, in a letter addressed to the editor of the *Lancet.*

That others, also, were of like opinions, may be proved by consulting the same journal for the ensuing month, as there may be found a letter from Mr. D. Mackenzie, entirely agreeing with the first writer. But it is very evident that these epistles did not produce results in harmony with the desires of the writers, for the surging wave of passing events threw nothing upon the shore which could be made available, and it was not until September, 1856, that we find anything tangible evolved. The "British Journal of Dental Science" contained in its second number (August, 1856) a letter from Mr. Rymer, in which he reiterated his views, and concluded by expressing his readiness to co-operate with his professional brethren in the formation of a Society of Dentists. The following number of the Journal contained an advertisement, announcing Mr. Rymer's intention of calling a public meeting of the profession, on the 22nd of September, at the London Tavern, Bishopsgate street, and asking for the attendance of such as sympathized with the contemplated projects.

The result of this advertisement was a copious correspondence on the subject, evincing a general desire that the object the advertiser had in view might be carried out, but in some instances, (as was expected,) depressing doubts and prophesies of ignominious failure, were by no means forgotten. Selecting the names of those who appeared most energetic in the cause, Mr. Rymer requested their attendance at a preliminary meeting, to confer upon the most appropriate method of conducting the forthcoming assembly. This meeting was convened for Saturday, the 20th September, at the London Tavern; and those who answered to the summons, were as follows: Messrs. Perkins, (Hockley Hill,) Brindly, (Sheffield,) Smith, (Chatham,) Bradshaw and Bate, (Brighton,) and Rymer.

Mr. Perkins, as the senior person present, occupied the chair. Such arrangements as were deemed necessary were then agreed upon, and this, the first meeting of gentlemen, (who were, with two or three exceptions, unknown to each other,) passed off with a spirit of cordiality and thorough good will, such as might have been expected only from and among friends of many years' standing—in itself a type and pledge of what would follow.

Those gentlemen, then, who formed this little convention, had ample cause for congratulation; for although in itself unostentatious enough, it was *the first stone* in the high wall of isolation loosened and thrown down—the *germ* of brotherly feelings, which was destined in its growth to eradicate the ancient spirit of jealousy and distinction, which so highly colored and completely pervaded the dental profession.

The experiment of testing the views of dental practitioners was now about to be performed; and the two short days which intervened were days of anxiety to the few more immediately concerned. At length the eventful Monday evening came; clad in wet garments, and with a look, dismal enough to dispirit and becloud the hopes of men depending upon far more substantial and united patrons for support, than those who now more anxiously than ever, awaited the result of the notice publicly advertised, of this their introductory meeting. The time wore on, and one by one (despite the wind and weather) the audience increased, until the room was *well filled;* some of those present having come very long distances, to testify in person to the necessity of professional reform and education. The gentlemen who had met on the previous Saturday, had decided that three concise, yet comprehensive propositions, should be laid before the meeting. Several copies of these had been printed, but it was necessary to procure movers and seconders, and this Mr. Rymer succeeded in effecting, by making personal application to several parties present. At the hour announced, Mr. Alfred Carpenter, M. B., was proposed and unanimously elected to fill the chair. This gentleman in his address explained that "he was not a dentist, but as a medical practitioner, he took a lively interest in the movement;" and as an impartial person, no doubt the selection of a gentleman out of the profession (so to speak) was a judicious one, in the absence of a dentist of the highest standing.

Mr. Rymer (who was warmly received) explained the reason which induced him to undertake the responsibility of convening the meeting, and proceeded to show why it was important that a step like the present should be taken. The three resolutions were then passed; the tendency of which was, that—1st, Reform in the profession was necessary; 2d, That immediate steps should be taken to produce that reform; and, 3d, That a committee to consider the best means of carrying out these ideas, should at once meet for that purpose.

The speakers on the occasion were Mr. Thomson (Denmark-hill) and Mr. Peter Matthews, as mover and seconder of the first resolution; Mr. Makenzie and Mr. Alfred Hill, as mover and seconder of the second; and Mr. Hockley and Mr. Perkins, as mover and seconder of the third. The meeting was addressed by other practitioners, and the unanimity and enthusiasm which prevailed, must in itself have been a reward to all who had a share in the matter.

The third resolution, which was as a matter of course the crowning one, involved little trouble to the parties proposing it. Without it, the two former ones would have been null and void; and, therefore, as soon as the bare formality respecting it had been gone through, in the most liberal manner imaginable, the names of certain gentlemen were put forward from the body of the meeting, and received, for the purpose of forming the committee. These were as follows:

Messrs. J. Bate, S. Cartwright, C. J. Fox, A. Hill, S. L. Jacob, D. Makenzie, Bradshaw, Coker, W. Harnett, Hockley, A. Lowe, P. Matthews, W. Perkins, S. L. Rymer, C. Stokes, R. Thomson, R. White, Rogers, E. Saunders, J. Jones, Watt, C. Vasey, with power to add their number.

Letters were read during the proceedings from Mr. Charles Stokes, Mr. F. J. C. Scott, Mr. White, and Mr. Edwin Saunders, all expressing approval in the movement.

A letter also was read from Mr. Thomas Bell, declining to take part in the affairs of the evening; and it must be here stated, that the offer of *presiding* had been made to this gentleman, which had also been declined.

Toward the latter part of the meeting, a statement was made by a gentleman present, that certain practitioners had taken up the idea of professional reform; and to this end had memorialised the council of the Royal College of Surgeons, praying for a special examination in dental surgery, as in midwifery.

This announcement created a degree of surprise, on account of the *privacy of the act*, and its consequent reflection upon the profession generally. The hitherto uncared for members of this branch of surgery awoke to the astonishing reality, that a small number of their body had gratuitously and secretly become the foster parents of the rest; and had actually taken steps in the direction of tutoring them as to the *only* means of becoming recognized and qualified practitioners of dentistry.

That the Royal College of Surgeons could not grant a petition *thus* presented, seemed patent to all present; and the irritating tendency of this effort was subdued by a seemingly general persuasion, that it would never realize the hopes of the petitioners; and so with the barest comment, this startling intelligence faded and died out.

The provisional committee elected, Mr. Rymer was requested to act in the capacity of secretary. To this he assented, but stated, that as his time was much occupied and his health not strong, he should require the assistance of a colleague. Whether or not this observation was forgotten by the audience it is not easy to say; but it is very *certain* that *no one* offered to share the difficulties and duties of this now important post; and it is, therefore, but an act of simple justice to Mr. Hill, who has so assiduously maintained that position, to say that he patiently waited until the assembly was about to disperse before he offered his services, which were most readily accepted by Mr. Rymer.

Thus, then, the men were supposed to have been found, who, for the love of their profession, and an earnest desire for its more extended usefulness, were ready to devote a portion of their influence and time to effect the organization of an honorable and scientific pursuit; and render the persons engaged in it a more amalgamated and less jealous body. The observations of the speakers (in one direction at least) pointed to the hope, that before long an institution would be established, with professorships and such other offices attached, as would tend to impart a dignity to all connected with it. The names of those practitioners, who by their scientific researches and discoveries, had deservedly received the undivided admiration of the entire profession, were brought forward as those who would best grace the professors' chairs; and not a few present on that eventful occasion congratulated themselves that there were gentlemen on the committee, to whom they might look with a full assurance of hope. The object was of the highest importance; the effort to obtain it most laudable; a little help from such as had wealth and position and acknowledged influence, was as natural for the one to look for as the other to give. True it was, that the professional dignitaries were placed above the necessity of alterations; true, again, that that elevated position enabled them to smile at the less fortunate practitioners, toiling up the hill, surrounded with encumbrances and disappointments; but no less true, the simple expectations that these same individuals, who, by force of circumstances singularly fortuitious, in days when competition in the dental community (then but small) was unknown, had gotten for themselves the lion's share, would *now* exert a little finger's strength to rectify a multitude of glaring errors. Unfortunately for these hopeful, hoping ones, the so-called leading men in the profession were not present in person; but yet they could not doubt the result of this public appeal to their generous sympathies,—they had only to be made acquainted with the great desire evinced on the occasion, to concur and blend their energies with the common cause.

Certainly, the meeting was called at the instigation of a non-metropolitan member of the profession; but surely it could not be a matter of consequence *who* set the ball of reform in motion, so long as it *was* set in motion. Certain, again, that the convention had not been announced by sound of trumpet, but it was so announced, that a hundred out of those who came to know of it, assembled at the prescribed place and hour. An opportunity was then given for *any* member of the profession, gentle or simple, to be present; and those who either could not or would not come, must not grumble that things went on without them. Here was an open meeting, to *discuss merely* the best method of making the wretched state of affairs, which was acknowledged by all to exist, better. The projector of the meeting, like many more at that moment, knew only a few practitioners personally, and a few more by other means; and to these invitations were sent, that they might come and assist in the deliberations of the evening. As before mentioned, Mr. Thomas Bell was invited to preside, evincing on the part of Mr. Rymer, a very laudable desire to have the benefit of that gentleman's matured judgment, to guide the opinion and resolutions which would be put forth on the occasion; unfortunately, however, for reasons of his own, the offer, as before stated, was declined.

Had any of the leading members of the profession appeared, there was all the opportunity that could be desired to mould the materials then in hand into another form; and certain beyond doubt it is, that Mr. Rymer would willingly have submitted to the *better* judgment of any of them who might have come forward; but as none of them did so, the parties then present combined to do for themselves what, if they chose, others might have done for them; and, although stigmatized as "*democratic, (?)*" as the meeting has since been, that part of the profession so comprehensive and vast, desiring alteration and improvement, will have no occasion to find fault with the materials employed to effect it.

Thus far Mr. Rymer had acted in accordance with views he had entertained for some years; and the success which up to this point attended his effort, was a precursor of similar success in future; when having gathered others round him, that movement which he had set in motion, but *single-handed could not keep in motion*, should progress and develop itself, attended by the good wishes of every true friend to the dental profession.

That mighty power, "THE SPIRIT OF THE AGE," demanded *reform;* and as now shown, it did not ask in vain.

The following is the "declaration" made by gentlemen on their admission as members of the college:

"In consideration of my being admitted a member of the College of Dentists of England, I hereby undertake and agree never to insert any advertisement or advertisements (excepting such as may be approved of by the council of the college) in any paper; nor to circulate handbills, exhibit show cases, boards, or placards, or to follow any other profession, business, or calling, whilst practicing as a dentist, or to do anything that may be in opposition to the 8th and 9th laws of the said college; and in the event of my departing from this agreement, I authorise the council of the said college to advertise my name in the public papers, as being no longer a member of the College of Dentists of England, immediately upon which I agree that my subscription or subscriptions shall become forfeited to the college."

(Signed)

The following speaks for itself:

"*College of Dentists of England,*
"5 *Cavendish Square, W.*

"SIR—The attention of the council having been called to your mode of practice, I am desired to inform you, that inasmuch as they consider such to be in opposition to the 8th and 9th laws of the college, they wish to know whether you are ready to sign the enclosed declaration; as by these laws, all members pursuing a similar course are no longer eligible to be members of the college. Awaiting your reply,

"I am sir, yours, faithfully,
"ALFRED HILL,
"*Hon. Corresponding Secretary.*

"P. S. Should you not return an answer to the above, within a fortnight from this date, the council will consider that you decline to sign the declaration.

